# Optimization for the Contrary-Rotating Double-Screw Extrusion of Plastics

**DOI:** 10.3390/polym15061489

**Published:** 2023-03-16

**Authors:** Andrzej Nastaj, Krzysztof Wilczyński

**Affiliations:** Polymer Processing Department, Faculty of Mechanical and Industrial Engineering, Warsaw University of Technology, Narbutta 85, 02-524 Warsaw, Poland; andrzej.nastaj@pw.edu.pl

**Keywords:** polymeric materials, counter-rotating twin-screw extrusion, optimization

## Abstract

A novel computer optimization system for the contrary-rotating double-screw extrusion of plastics was developed in this study. The optimization was based on the process simulation performed with the use of the global contrary-rotating double-screw extrusion software TSEM. The process was optimized using the GASEO_TWIN_ software developed for this purpose using genetic algorithms. Several examples of optimization of the contrary-rotating double screw extrusion process parameters, i.e., the extrusion throughput, and minimize the plastic melt temperature and the plastic melting length.

## 1. Introduction

Nowadays, computer simulations facilitate the development of polymer processing techniques and enable the prediction of the process course based on process parameters, i.e., material data, geometry data, and operating data. Simulations, however, do not allow researchers to conduct this process in reverse, i.e., to compute the process parameters to obtain the optimal output parameters.

Extrusion is the most used mass technique in the polymer processing industry. It is used for manufacturing films, sheets, pipes, and profiles, as well as for specialized operations, e.g., for compounding, reinforcing, pelletizing, etc. Extruders are divided into single-screw and double (or twin)-screw machines. Double-screw machines can be co-rotating or contrary-rotating (or counter-rotating).

Underwood [1] and Verbraak and Meijer [2] were the first to perform experiments to optimize extrusion. The main drawback of this approach is the number of experiments needed. Optimization by experiments is time-consuming, expensive, and nothing warranted that global optimum has been found.

Therefore, process simulations with statistical support seem to be better. The first optimizations using simulations were performed by Tadmor and Klein [3], Maddock and Smith [4], as well as by Helmy and Parnaby [5]. Later, Potente and Krell [6] used the REX software [7,8,9], Thibodeau and Lafleur [10,11] used the software of Ecole Polytechnique de Montreal [12,13], and the authors used the SSEM (Single Screw Extrusion Model) software. The drawback of these statistical approaches was the number of simulations needed, and a danger of selecting the local optima.

Covas and Gaspar-Cunha proposed a new strategy for extrusion optimization based on genetic algorithms. They developed procedures for the optimization of single-screw extrusion [14,15,16] and later for co-rotating double-screw extrusion [17,18,19]. The authors also used genetic algorithms for the optimization of single-screw extrusion [20]. The concept of genetic algorithms was also applied for scaling up the extrusion [21,22], as well as for the optimization of injection molding [23,24]. Nastaj and Wilczyński developed the optimization procedures for starve-fed single-screw extrusion [25,26] using the original models of this process [27,28,29,30].

Genetic algorithms are characterized by the following features:

- Optimization variables are coded;
- Searching for a solution starts from some population, which means the probability of getting stuck at a local extreme is low;
- The rules of selection are probabilistic;
- Objective functions are used, and derivatives are not necessary.

So far, there is a lack of optimization procedures for contrary-rotating double-screw extrusion, although computer models of this process have been recently proposed [31,32,33].

In this paper, a novel computer optimization system for the contrary-rotating double-screw extrusion of plastics was developed. Optimization was based on the process simulations performed with the use of the global contrary-rotating double-screw extrusion software TSEM [34]. The process was optimized using the GASEO_TWIN_ software developed for this purpose using genetic algorithms. Several examples of the parameter optimization of the contrary-rotating double-screw extrusion process were studied in order to maximize the flow rate, i.e., the extrusion throughput, and minimize the plastic melt temperature and the plastic melting length, which are presented in the following sections.

## 2. Contrary-Rotating Double-Screw Extrusion

**Figure 1:**
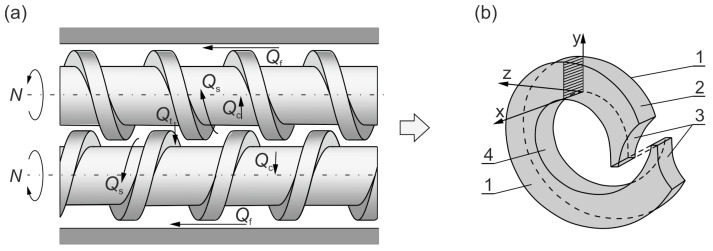
The plastic flow in a contrary-rotating double-screw extruder: (**a**) leakage flows, Q_c_—calendering flow, Q_f_—flight flow, Q_t_—pressure (tetrahedral) flow, Q_s_—side flow; (**b**) C-shaped chamber, 1—side surface of the screw flight, 2—barrel surface, 3—front surface of the screw flight, 4—surface of the screw root (adopted with permission from Wilczyński, K. *Rheology in Polymer Processing. Modeling and Simulation*; Carl Hanser Verlag: Munich 2021 [34]).

Double (or twin)-screw extrusion is divided into co-rotating extrusion (the screws are rotating in the same direction), and contrary-rotating (or counter-rotating) extrusion (the screws are rotating in the opposite direction (Figure 1)).

Contrary-rotating double-screw extruders are mainly applied for processing thermally sensitive plastic, e.g., polyvinyl chloride (PVC). Co-rotating double-screw extruders have specialized applications, e.g., for compounding, mixing, filling, and reinforcing plastics. The scheme of contrary-rotating double-screw extrusion is depicted in Figure 1.

Contrary-rotating double-screw extruders, compared with single-screw extruders, provide better feeding capability, as they can feed the machine with materials in the form of powder or with materials exhibiting slip properties. In contrary-rotating extruders, the material essentially does not flow from one screw to the other, as in co-rotating extruders. In the case of contrary-rotating machines, there is a co-rotating movement in the inter-screw gap, so high shear stresses are not produced in this area, as in co-rotating machines.

Contrary-rotating extruders are usually fed with dosing (metered feeding or starve feeding). The flow of plastics in these machines is completely different from the flow in the single-screw and co-rotating extruders. This flow results from a positive displacement mechanism, which does not appear in other extruders. The degree of positive displacement is dependent on the degree of screw meshing. It takes place most fully in closely intermeshing counter-rotating machines.

The material in the contrary-rotating extruder was transported in a C-shaped chamber (Figure 1b), and some leakage flows were also observed (Figure 1a), i.e., the calendering flow Q_c_, the flight flow Q_f_, the pressure (tetrahedron) flow Q_t_, and the side flow Q_s_. The C-chamber was formed by six surfaces, namely the screw root surface, the inner barrel surface, the side surfaces of the screw flights (twice), and the front surfaces of the screw flights (twice). There were distinguished leakage flows between these surfaces, i.e., the calendering flow Q_c_ between the screw root and the screw flight; the flight flow Q_f_ over the screw flights, through the clearance between the screw flight and the barrel; the back pressure inter-screw flow Q_t_ (the tetrahedron flow) through the tetrahedral clearance between the flight flanks of screws, in the radial direction; and the side flow Q_s_ through the side gap between the flight flanks of screws, in the tangential direction.

## 3. Modeling of Contrary-Rotating Double-Screw Extrusion

Some fundamental books present the state of the art in the modeling of polymer extrusion, e.g., [35,36,37,38,39], as well as some papers, e.g., [40,41,42,43]. Wilczyński et al. summarized this body of research in a review paper [44].

The fundamentals of contrary-rotating double-screw extrusion were first discussed a hundred years ago [45,46,47,48], and many designs of these machines were later developed [49,50].

Contrary-rotating extruders are entirely different from single-screw extruders, as well as from co-rotating machines. They were first presented by Kiesskalt [46] and Schenkel [49] as positive displacement pumps. Doboczky [51] and Janssen [52] proposed the flow-pumping characteristics of these extruders, and they considered the leakage flows. White and Adewale [53] performed the modeling of the flow in these machines while taking the level of screw intermeshing into consideration. Numerical FEM simulations were carried out by Li and Manas-Zloczower [54] and by Kajiwara et al. [55]. Hong and White [56,57] developed a FAN analysis for the non-Newtonian flow in these machines. They presented the concept of screw characteristics, which enabled them to model the flow for various screw designs and compute the pressure, fill factors, and temperature profiles.

Investigations of solid plastic conveying and plastic melting in contrary-rotating machines are very limited [51,52]. Wilczyński and White were the first who experimentally investigated and modeled the melting process [58,59]. Further studies were presented by Wang and Min [60,61] and by Wilczyński et al. [62].

White et al. [56,57,58,59] developed a theory that allowed for the prediction of both the pumping capacity and plastic melting profiles in these machines. The first model of this process was presented by Wilczynski et al. [31] to predict solid conveying, melting, and melt flow. Jiang et al. [63] developed a model for contrary-rotating double-screw extruders with flood feeding.

Lewandowski et al. [33] proposed an integrated model for the entire double-screw extruder, including the die. This model is based on combining melt-conveying models with melting and solid-conveying models. Three-dimensional, non-Newtonian FEM computations for melt conveying were performed to determine the pumping characteristics of the screws, which were included in the global model. To our knowledge, this was the first and, up to now, the only available global model of contrary-rotating double-screw extruders based on three-dimensional non-Newtonian FEM computations. This model was later extended, and it is now part of the software called Multi-Screw System [34].

The model we used in the studies presented here was based on our previous experimental investigations, which were discussed in the literature [58,62]. In these investigations, the “screw pulling-out technique” was applied to observe the plastic flow along the screws. The screws were removed from the barrel, the plastic contained in the screw channel was estimated, and the plastic samples were scrapped off from each screw. In the hopper section, there were solid granules (pellets) freely transported along the screws. The pellets were collected at the bottom part of the barrel adjacent to the pushing flights of the screws. The pellets were heated with the heat from the barrel and by being dragged into the gap between the screws. They formed some kind of solid bed, which decreased in length along the screws. The solid bed moved along the screw as a part of the C-chamber, and it was dragged into the calendering gap. High pressure was developed in the gap region, where friction forces initiated the melting process. Afterward, melting proceeded from the hot barrel, and a melt layer was formed between the barrel and the solid bed, which was scrapped off using the screw flights. The molten plastics flowed in a starved manner. The area close to the die was pressurized, and the screw became fully filled with plastic. Finally, the plastic flowed through the die under the pressure developed in the screw channel.

Based on these observations, models were developed for melting in both those regions, i.e., in the calendering gap and in the melt layer. Melting was initiated between the screws by the friction on the pellets, which was caused by the calendering stresses between the screws. The melting action at the barrel was induced by the barrel temperature being higher than the melting point and was propagated by the viscous dissipation heating of the melt film produced.

The model we used was experimentally validated for various screw configurations, various operating conditions, and various materials, namely polypropylene (PP), low-density polyethylene (LDPE), and high-density polyethylene (HDPE). This was presented in the literature, e.g., [31,33,34,59].

**Figure 2:**
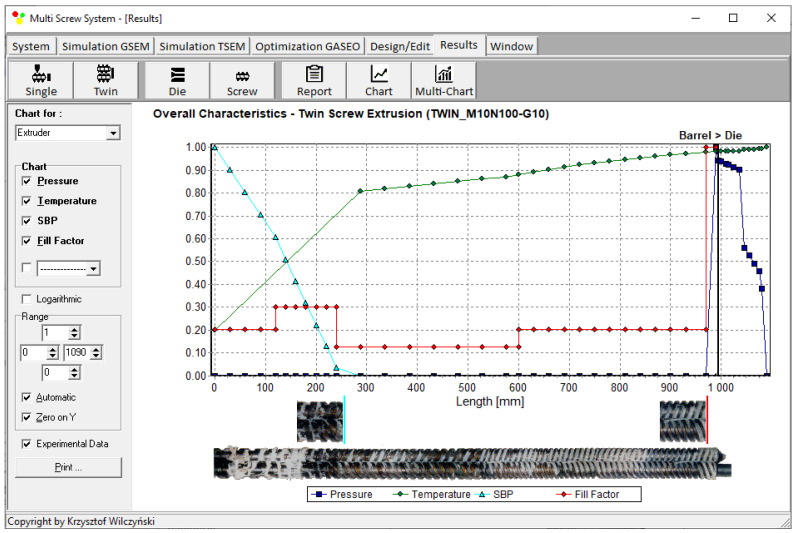
General extrusion characteristics, simulation data using TSEM model—twin-screw extrusion, Q = 10 kg/h, screw speed N = 100 rpm: SBP—solid-bed profile.

**Figure 3:**
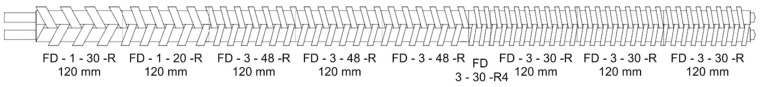
Screw configurations.

An example of computations with the use of TSEM software (and experimentation) is shown in Figure 2 for the screw system depicted in Figure 3. The experiment was performed for the extrusion of polypropylene (PP) with the screw speed of N = 100 rpm and the mass flow rate (throughput) of G = 10 kg/h. The dimensionless extrusion characteristics are presented with pressure (P—navy blue line) and temperature (T—green line) profiles, while the profile of plastic melting is represented by the solid-bed profile (SBP—blue line), and the profile of screw filling is represented by the fill factor (FF—red line). As can be seen, the pressure was only built in the region of fully filled screws. It is also interesting to note that the screws were fully filled with the material only at the final short section before the die. In the rest of the extruder, the screws were only 10–30% filled.

**Figure 4:**
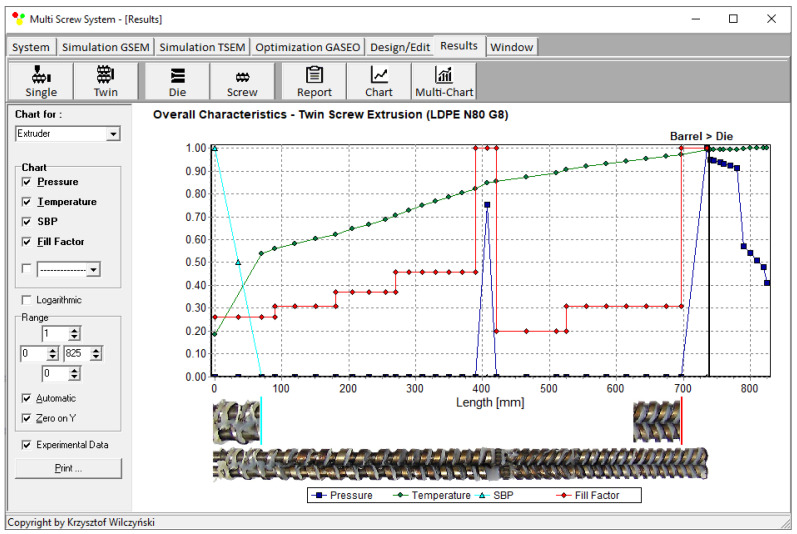
General extrusion characteristics, simulation data using TSEM model—twin-screw extrusion, Q = 8 kg/h, screw speed N = 80 rpm: SBP—solid-bed profile (adopted with permission from Wilczyński, K. *Rheology in Polymer Processing. Modeling and Simulation*; Carl Hanser Verlag: Munich 2021 [34]).

**Figure 5:**
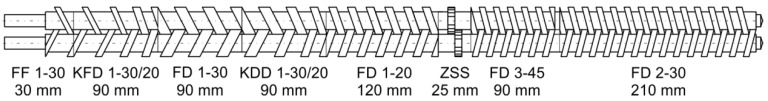
Screw configurations (adopted with permission from Wilczyński, K. *Rheology in Polymer Processing. Modeling and Simulation*; Carl Hanser Verlag: Munich 2021 [34]).

Another example of computations with the use of TSEM software (and experimentation) is shown in Figure 4 for the screw system depicted in Figure 5. This experiment was performed for the extrusion of low-density polyethylene (LDPE) at the screw speed of N = 80 rpm with the mass flow rate (throughput) of G = 8 kg/h. In this case, it is also seen that the pressure is built only in the region of fully filled screws. However, the screws were also fully filled with the material in the region of shearing elements (ZSS), and the pressure also developed in this region. In the rest of the extruder, the screws were only 20–30% filled.

## 4. Optimization Procedure

The GASEO_TWIN_ software (Genetic Algorithm Screw Extrusion Optimization) has been developed to solve the optimization issue. Simulations with the use of the TSEM (Twin-Screw Extrusion Model) software [44] were the source of optimization data. The GASEO_TWIN_ software is characterized by TSEM-specific data exchange between the simulation program and the optimization program.

The GASEO_TWIN_ software, combined with the TSEM simulation software, enables process optimization with any number of optimized parameters and various criteria of optimization criteria. The accuracy of searching for the optimized response surface is determined by the number of divisions of the data range, which results from the length of the series of these numbers in a binary form. In GASEO_TWIN_ software, the length of binary series is adjustable, and its maximum length is equal to 255 characters.

**Figure 6:**
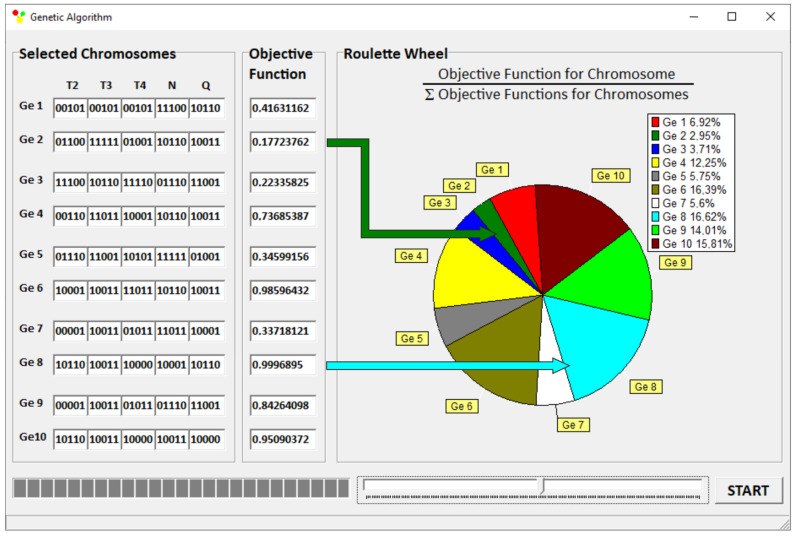
A “roulette wheel”.

A “roulette wheel” concept was used for selection with an elitist strategy (Figure 6). In this strategy, the best dataset automatically continues to be used in the next generation, protecting the algorithm from losing the dataset with the highest value of objective functions. The algorithm stops if there is no improvement in the value of the objective function for 100 iterations. An action of the “roulette wheel” is presented in Figure 4. The areas of the “roulette wheel” defining the genotypes were proportional to the objective functions determined by these genotypes. As an example, the genotype Ge8 yielded the highest objective function F_8_ = 0.9996 and occupied a surface equal to 16.62% of the entire surface of this “roulette wheel”. The genotype Ge2 had the lowest objective function F_2_ = 0.1772, occupying a surface equal to 2.95% of the entire surface of the “roulette wheel”.

The optimization procedures were determined using GA parameters, i.e., the number of optimized variables, the size of the initial population, the length of chromosomes, the probability of crossover and mutation, and the points of crossover.

## 5. Optimization

### 5.1. Research Program

Optimization was performed for the contrary-rotating double-screw extrusion of polypropylene (PP). The calculations were carried out for a Lesitritz LSM 30/34 modular intermeshing contrary-rotating double-screw extruder. This is the machine intended for compounding. We considered the flow in the screw configurations depicted in Figure 3. The screws had a 34 mm diameter with a 30 mm distance between the center lines.

Polypropylene (PP) has a density of 0.904 g/cm^3^ (solid) and 0.739 g/cm^3^ (melt), and a melt flow rate (MFR) of 2.7 g/10 min (230 °C, 2.16 kg). The rheological flow properties of PP were determined on the basis of capillary rheometry and modeled using the Klein equation as follows:

(1) $$ln\eta =A_{0}+A_{1}ln\dot{\gamma }+A_{11}ln^{2}\dot{\gamma }+A_{12}Tln^{2}\dot{\gamma }+A_{2}T+A_{22}T^{2}$$

where η is the viscosity; $\dot{\gamma }$ is the shear rate; T is the temperature; and *A*_0_, *A*_1_, *A*_11_, *A*_12_, *A*_2_, and *A*_22_ are the model parameters (*A*_0_ = 14.0587, *A*_1_ = −0.4535, *A*_11_ = −0.0281, *A*_12_ = 2.71 × 10^−4^, *A*_2_ = −0.0316, and *A*_22_ = 4.45 × 10^−5^).

Optimization was carried out to maximize the extrusion output *Qmax* (kg/h), minimize the plastic temperature at the die outlet *T_out_* (°C), and minimize the plastic melting length *L_pl_* (mm). Contrary-rotating double-screw extruders are often used for the extrusion of thermally sensitive polymers; thus, minimizing the plastic temperature is important. Moreover, minimizing the temperature means minimizing power consumption. Minimizing the plastic melting length provides a sufficiently large flow space for a good mixing process of the plasticized material.

The optimized parameters were the screw rotation, the cylinder temperatures, and the extrusion throughput. These are presented in Table 1, where their range is also shown. Optimization was carried out for various weighted criteria, which were divided into two groups. In the first group (Table 2, items 1, 2, and 3), there was one criterion with a dominant weight (*w_i_* = 0.8), whereas in the second group (Table 2, items 4, 5, and 6), there were two criteria with both dominant and equal weight (*w_i_* = 0.4).

The global objective function is defined as

(2) $$F_{i}=w_{Q}\cdot Q_{i{\_}_{norm}}+w_{Tout}\cdot T_{out\;i\_norm}+w_{Lpl}\cdot L_{pl\;i\_norm}$$

where the output parameters (optimization criteria) are normalized as

(3) $$Q_{i\_norm}=\frac{Q_{i}-Q_{min}}{Q_{max}-Q_{min}}$$

(4) $$T_{out\;i\_norm}=\frac{T_{out\;max}-T_{out\;i}}{T_{out\;max}-T_{out\;min}}$$

(5) $$L_{pl\;i\_norm}=\frac{L_{pl\;max}-L_{pl\;i}}{L_{pl\;max}-L_{pl\;min}}$$

where *F_i_* is the global objective function, *Q_i_norm_* is the normalized flow rate (extrusion throughput), *w_Q_* is the weight of flow rate, *T_out i_norm_* is the normalized plastic temperature at the die outlet, *w_Tout_* is the weight of plastic temperature at the die outlet, *L_pl i_*_*_norm*_ is the normalized plastic melting length, *w_Lpl_* is the weight of plastic melting length, and *i* is the number of the next value from the dataset.

### 5.2. Results

The optimization results are presented for various weights of the optimization criteria in Table 2.

Based on the obtained results, it can be concluded that, despite the different weights of the optimization criteria, the most common solutions were the sets of operating parameters that were similar in value (Table 2, items 1, 2, and 4). They had the following values: N = 227.5 rpm, T_II_ = 180 °C, T_III_ = 180 °C, T_IV_ = 180 °C and 187.5 °C, and Q = 65.63 kg/h and 62.50 kg/h. The optimization results showed that the operating parameters of the process both in the case when the highest weight was assumed for the mass flow rate (extrusion throughput; *w_Q_* = 0.8) and in the case when the highest weight was assumed for the lowest temperature of the material at the outlet of the die (*w_Tout_* = 0.8) were also close together. It follows that the selection of either of these sets of operating parameters (Table 2, items 1 and 2) allows us to obtain the course of the extrusion process with relatively the highest throughput and the lowest plastic melt temperature.

**Figure 7:**
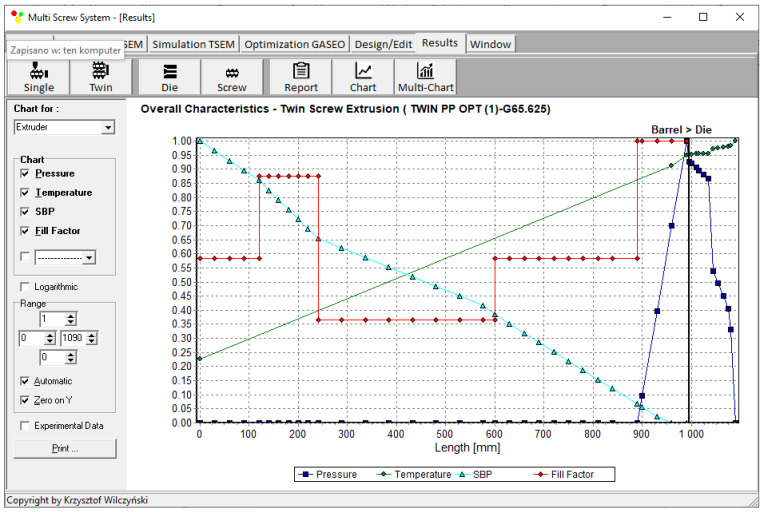
Extrusion characteristics (weights of optimization criteria: *w_Q_* = 0.8, *w_Tout_* = 0.1, and *w_Lpl_* = 0.1).

**Figure 8:**
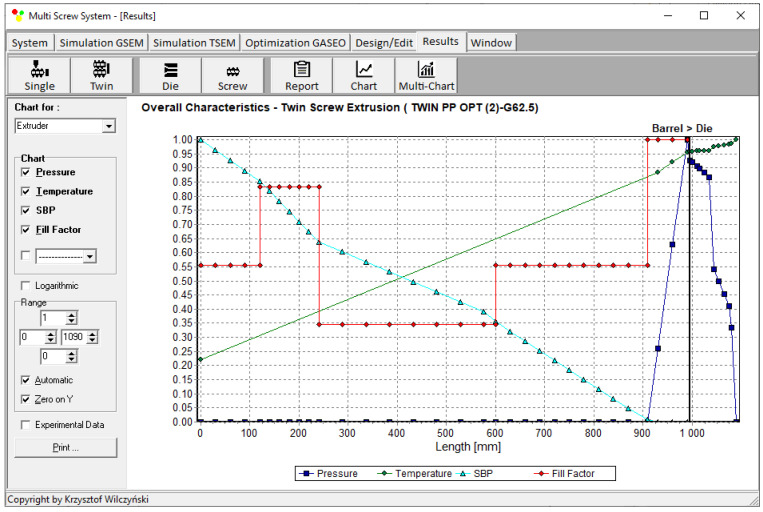
Extrusion characteristics (weights of optimization criteria: *w_Q_* = 0.1, *w_Tout_* = 0.8, and *w_Lpl_* = 0.1).

**Figure 9:**
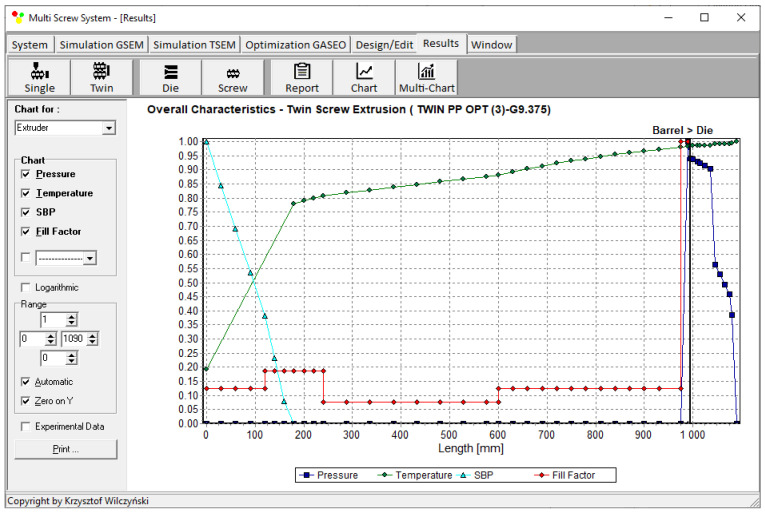
Extrusion characteristics (weights of optimization criteria: *w_Q_* = 0.1, *w_Tout_* = 0.1, and *w_Lpl_* = 0.8).

**Figure 10:**
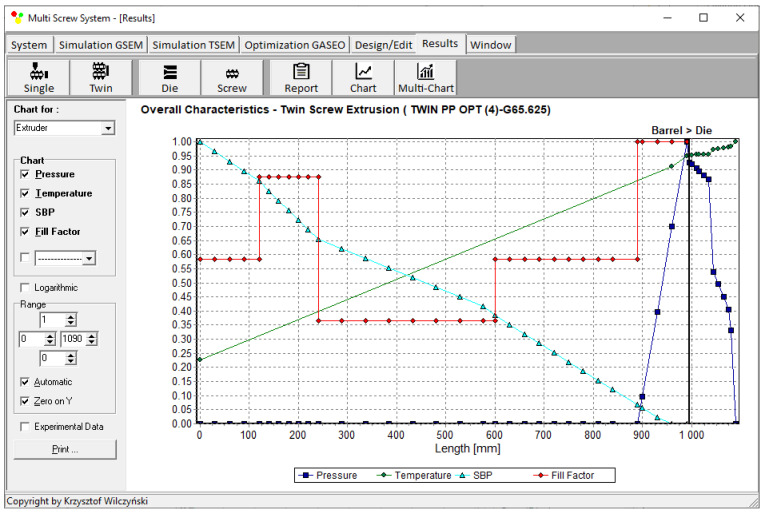
Extrusion characteristics (weights of optimization criteria: *w_Q_* = 0.4, *w_Tout_* = 0.4, and *w_Lpl_* = 0.2).

**Figure 11:**
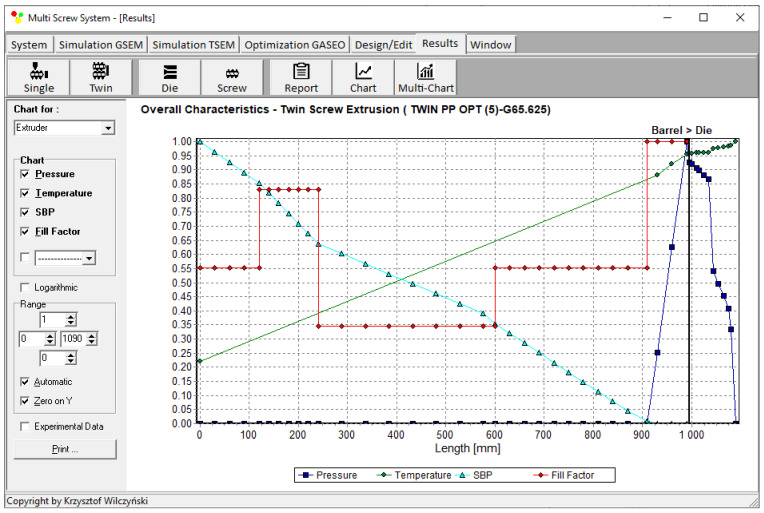
Extrusion characteristics (weights of optimization criteria: *w_Q_* = 0.4, *w_Tout_* = 0.2, and *w_Lpl_* = 0.4).

**Figure 12:**
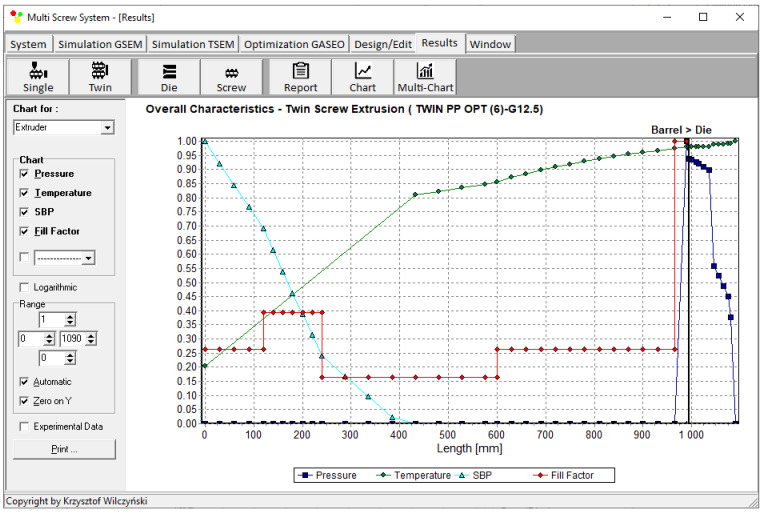
Overall process characteristics for twin-screw extrusion (weights of optimization criteria: *w_Q_* = 0.2, *w_Tout_* = 0.4, and *w_Lpl_* = 0.4).

**Figure 13:**
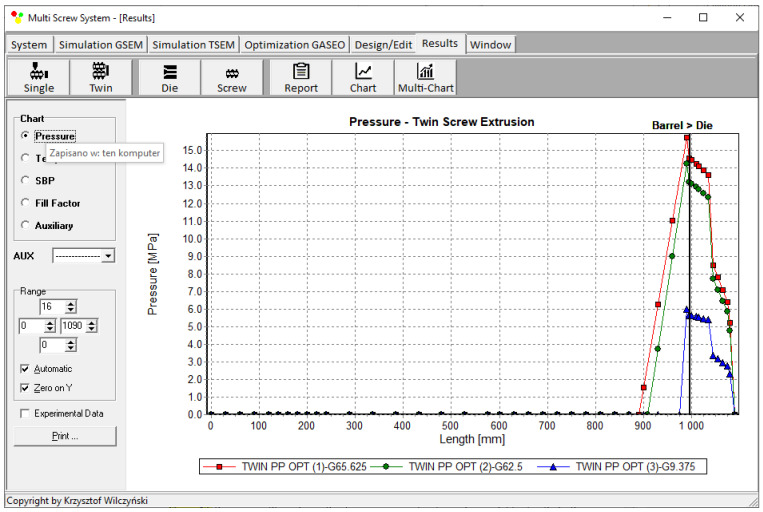
Pressure profiles using optimal process parameters for various weights of optimization criteria: *w_Q_*, *w_Tout_*, and *w_Lpl_* (Table 2, results 1–3).

**Figure 14:**
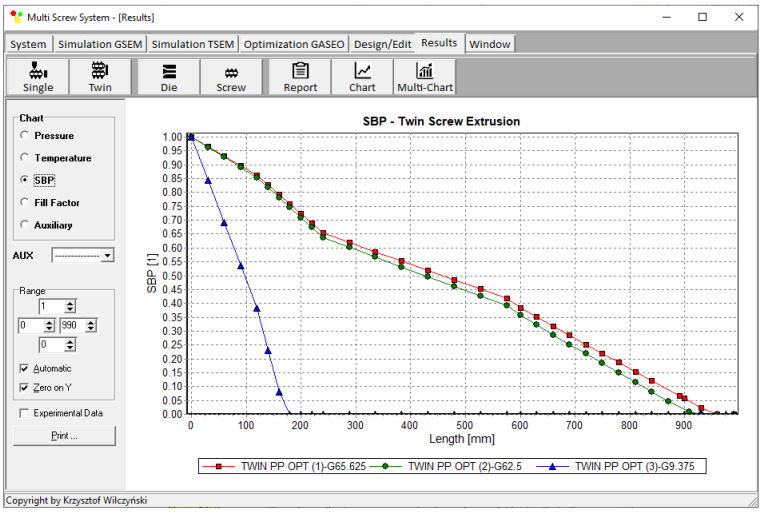
Solid-bed profiles (SBPs) using optimal process parameters for various weights of optimization criteria: *w_Q_*, *w_Tout_*, and *w_Lpl_* (Table 2, results 1–3).

**Figure 15:**
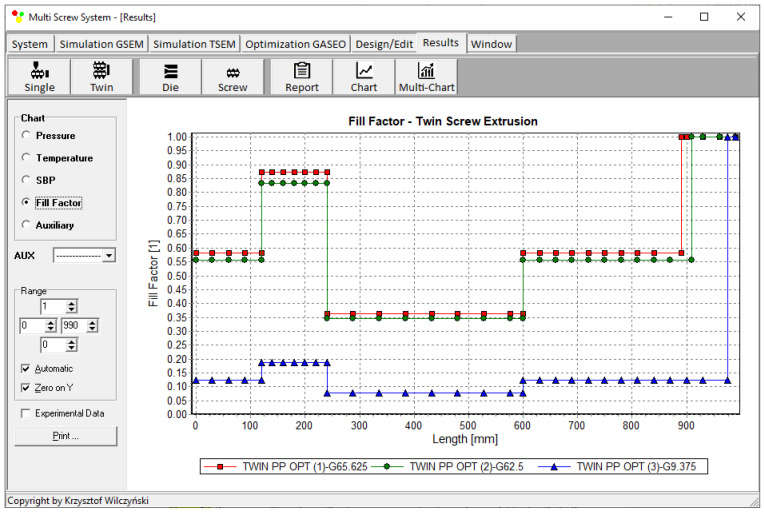
Fill-factor profiles using optimal process parameters for various weights of optimization criteria: *w_Q_*, *w_Tout_*, and *w_Lpl_* (Table 2, results 1–3).

**Figure 16:**
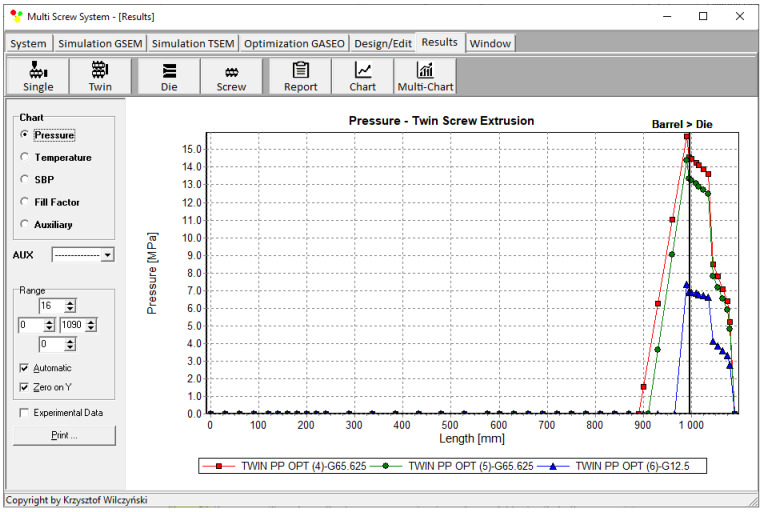
Pressure profiles using optimal process parameters for various weights of optimization criteria: *w_Q_*, *w_Tout_*, and *w_Lpl_* (Table 2, results 4–6).

**Figure 17:**
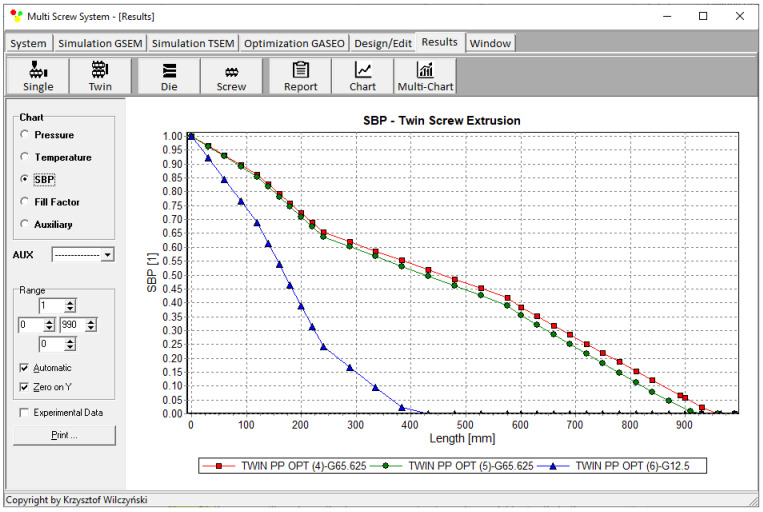
Solid-bed profiles (SBPs) using optimal process parameters for various weights of optimization criteria: *w_Q_*, *w_Tout_*, and *w_Lpl_* (Table 2, results 4–6).

**Figure 18:**
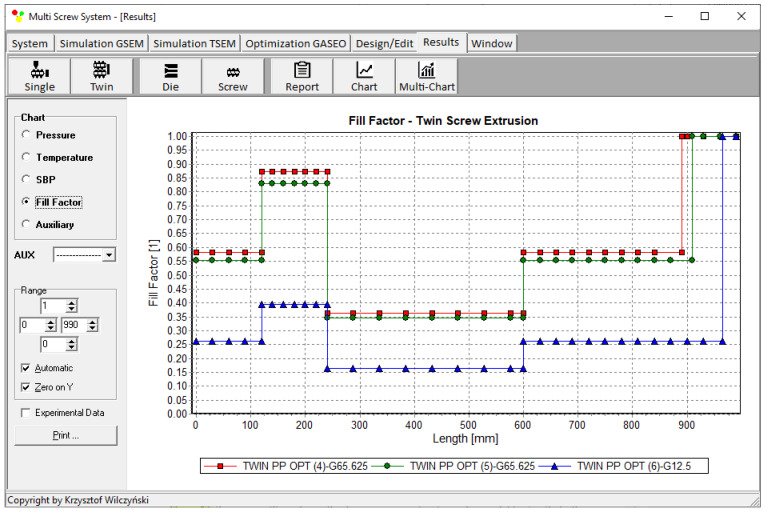
Fill-factor profiles using optimal process parameters for various weights of optimization criteria: *w_Q_*, *w_Tout_*, and *w_Lpl_* (Table 2, results 4–6).

Process simulations were performed for each set of optimal parameters (N, T_II_, T_III_, T_IV_, and Q). The simulation results using the optimal parameters for various weights of optimization criteria are presented in Figure 7, Figure 8, Figure 9, Figure 10, Figure 11, Figure 12, Figure 13, Figure 14, Figure 15, Figure 16, Figure 17 and Figure 18. The overall process characteristics (extrusion characteristics) obtained with the optimal parameters are depicted in Figure 7, Figure 8, Figure 9, Figure 10, Figure 11 and Figure 12, whereas the process variables, i.e., the pressure profiles (P—navy blue line), the temperature profiles (T—green line), the solid-bed profiles (SBP—blue lines), and the fill-factor profiles (FF—red line), using the optimal process parameters (N, T_II_, T_III_, T_IV_, and Q) are depicted in Figure 13, Figure 14, Figure 15, Figure 16, Figure 17 and Figure 18.

It is interesting to note that, as shown in Figure 11, Figure 12, Figure 13, Figure 14, Figure 15, Figure 16, Figure 17 and Figure 18, the simulation results for optimal sets (3) and (6) differed from other simulations. These simulations were carried out with the weights of *w_Q_* = 0.1, *w_Tout_* = 0.1, and *w_Lpl_* = 0.8 as well as *w_Q_* = 0.2, *w_Tout_* = 0.4, and *w_Lpl_* = 0.4, both indicating the cases in which the weight of the polymer melting length *w_Lpl_* had the dominating value.

**Figure 19:**
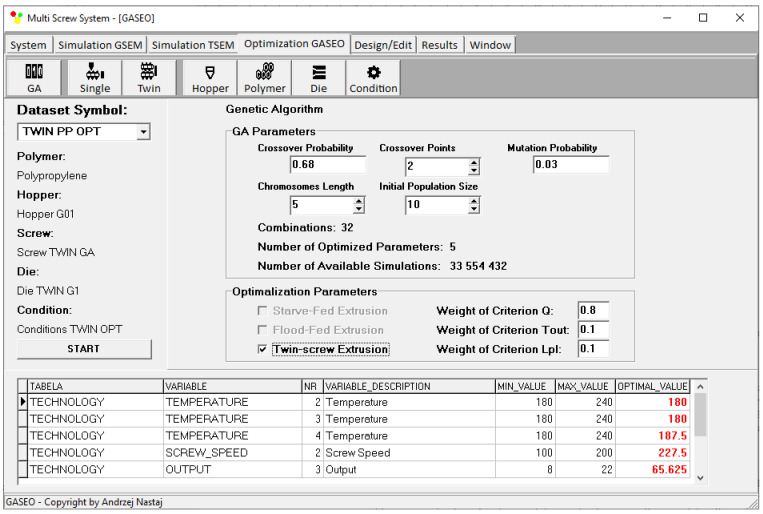
Optimization results for twin-screw extrusion for weights of optimization criteria equal to *w_Q_* = 0.8, *w_Tout_* = 0.1, and *w_Lpl_* = 0.1.

An example of the optimization results (for criteria weights *w_Q_* = 0.8, *w_Tout_* = 0.1, and *w_Lpl_* = 0.1) as shown on the screen of the GASEO_TWIN_ program is depicted in Figure 19. The parameters of optimization are also seen, as well as the values of optimal parameters.

## 6. Conclusions

A novel computer optimization system for contrary-rotating double-screw extrusion of plastics was developed. Optimization was based on the process simulation performed with the use of the global contrary-rotating double-screw extrusion software TSEM. The process was optimized using the GASEO_TWIN_ software, developed for this purpose using genetic algorithms.

An example of using this system was presented to show the possibilities of the developed system. Optimization was carried out in order to maximize the flow rate, i.e., the extrusion throughput, minimize the plastic melting temperature at the die outlet, and minimize the plastic melting length. These parameters are important when using double-screw extruders. Contrary-rotating extruders are often used for the extrusion of thermally sensitive plastics; thus, minimizing the plastic temperature is important. Moreover, minimizing the plastic temperature means minimizing power consumption. Minimizing the plastic melting length provides a sufficiently large flow space for the good mixing of the plasticized material. Additionally, these optimization criteria were used with various weights, which depend on the manufacturer’s requirements and are decided by the manufacturer. In this example, the optimized parameters were the screw rotation, the cylinder temperatures, and the extrusion throughput, that is, the basic operating parameters of the process. Of course, optimization can be also performed for the optimization of the geometry of screws.

To our knowledge, this is the first optimization software for contrary-rotating double-screw extrusion that uses process simulations. It enables the optimization of extrusion process parameters using various optimization criteria with various weights.

## Figures and Tables

**Table 1:** Research program.

Screw Rotation N, rpm	Cylinder Temperature T_I_, °C	Cylinder Temperature T_II_, °C	Cylinder Temperature T_III_, °C	Cylinder Temperature T_IV_, °C	Extrusion Output Q, kg/h
40 ÷ 240	180	180 ÷ 240	180 ÷ 240	180 ÷ 240	1 ÷ 100

**Table 2:** The objective functions (maxima) and the optimized parameters with various weights of optimization criteria.

	Criterion Weights					
	*w_Q_* = 0.8 *w_Tout_* = 0.1 *w_Lpl_* = 0.1	*w_Q_* = 0.1 *w_Tout_* = 0.8 *w_Lpl_* = 0.1	*w_Q_* = 0.1 *w_Tout_* = 0.1 *w_Lpl_* = 0.8	*w_Q_* = 0.4 *w_Tout_* = 0.4 *w_Lpl_* = 0.2	*w_Q_* = 0.4 *w_Tout_* = 0.2 *w_Lpl_* = 0.4	*w_Q_* = 0.2 *w_Tout_* = 0.4 *w_Lpl_* = 0.4
No	1	2	3	4	5	6
Screw rotation N, rpm	227.5	227.5	152.5	227.5	240	96.3
Cylinder temperature T_II_, °C	180	180	219.4	180	180	196.9
Cylinder temperature T_III_, °C	180	180	219.4	180	180	180
Cylinder temperature T_IV_, °C	187.5	180	230.6	180	187.5	187.5
Extrusion throughput Q, kg/h	65.63	62.50	9.38	65.63	65.63	12.50
Temperature of plastic at die outlet *T_out_*, °C	176.25	181.49	206.42	175.75	181.58	197.24
Length of plastic melting *L_pl_*, mm	960	920	160	960	920	240
Objective function *F_i_*	0.6243	0.8563	0.8373	0.6673	0.4934	0.5819

## Data Availability

The data presented in this study are available on request from the corresponding author.
